# Adjustable bone plate: from bench to operating room

**DOI:** 10.55730/1300-0144.5704

**Published:** 2022-10-10

**Authors:** Gazi HURİ, Erdi ÖZDEMİR, Ömer Sunkar BİÇER, Pınar YILGÖR HURİ, Sait POLAT, Tuğçe SAPMAZ, Mahmut Nedim DORAL

**Affiliations:** 1 Department of Orthopedics and Traumatology, Faculty of Medicine, Hacettepe University, Ankara, Turkiye; 2 Department of Orthopedics and Traumatology, Faculty of Medicine, Çukurova University Adana, Turkiye; 3 Department of Biomedical Engineering, Faculty of Engineering, Ankara University, Ankara, Turkiye; 4 Department of Histology and Embryology, Faculty of Medicine, Çukurova University Adana, Turkiye; 5 Department of Orthopedics and Traumatology, Faculty of Medicine, Ufuk University, Ankara, Turkiye

**Keywords:** Adjustable bone plate, bone defect, segmental bone transfer, trauma

## Abstract

**Background/aim:**

We have designed an adjustable bone plate (ABP) which allows bone shortening and lengthening after fixation, which is a property not present in any of the plate systems available today. The aim of the current study was to examine the new ABP’s segmental bone transfer capability for the treatment of a segmental bone defect in an animal model.

**Materials and methods:**

Five sheep had ABPs attached to 10 of their tibias and bone defects of 15 mm in size were created. The pinion mechanism was moved with a manual screwdriver at a rate of 1mm/day for 15 days starting 3 days postoperatively. The animals were euthanized 3 months postoperatively, and the defect site and the transferred segment were evaluated by radiological and histological examination.

**Results:**

The radiological results revealed successful transfers of 14.6 ± 1.2 mm of bone segment on all tibia defects without any complications. The histological evaluation showed new bone formation in both the extension and the docking sites. No rupture or breakage was observed within the plates.

**Conclusion:**

We have presented the potential of a new generation ABP for use in segmental bone transfer in an animal model as well as for future clinical applications.

## 1. Introduction

Orthopedic implants such as intramedullary nails, external fixators, plates, and screws are most frequently used for the surgical treatment of fractures [1–3]. Apart from fracture treatment, these implants can also be used for different orthopedic purposes, including deformity correction, treatment of limb length inequalities, and reconstructions following bone resections [4–7].

In the treatment of limb length discrepancies (LLD) and bone defects, several surgical techniques and devices have been developed [8,9]. Patients with such pathologies often experience pain, dysfunction, joint degeneration, and aesthetic problems [10]. The primary method of treating deformities such as bone defects and LLD is an osteotomy followed by callus distraction or segment transfer. Two commonplace types of lengthening device are external fixators and intramedullary devices, but each has its drawbacks [5]. Traditional external fixators, such as the Ilizarov device and other external fixators, are cumbersome, painful, and produce large residual scars. Lengthening with these methods requires careful monitoring due to pin tract infection rates of 10%–20% [11]. Furthermore, intramedullary lengthening devices can cause severe complications such as failure of the lengthening mechanism, migration of locking screws, and intramedullary infection. Surgeons have recently experienced success with a motorized intramedullary nail, but pediatric use of this device can be limited due to interference with open growth plates [12].

With the currently available designs and technologies, conventional plates cannot be used alone for segmental bone transfer or bone lengthening. The existing plates only allow up to 2 mm of bone movement in the fracture line and do not allow any further maneuvers [13]. Furthermore, after having been applied to the bone, conventional plates do not allow adjusting the fracture line; thus, a mispositioning (i.e. a nonabutment of fracture ends) requires a further procedure, lengthening the surgery period and increasing the risk of complications. Additionally, the state-of-the-art bone plates are of a static nature, thereby requiring the use of auxiliary elements such as external fixators, especially where a bone shortening or bone extension is necessary [14]This procedure requires further medical personnel or a second fixation implant, causing redundant labor loss, additional cost and needless application of an additional (albeit temporary) implant to the patient. Based on these requirements and knowledge, we have designed a novel adjustable bone plate (ABP) that replicates the attractive qualities of an expandable intramedullary nail and an external fixator device while reducing the risk of deep infection and causing no damage to growth plates. We hypothesized that a segmental bone transfer could be performed with this novel ABP. In the current study, we aimed to investigate the capability of the new ABP on the treatment of bone defects in an animal model.

## 2. Materials and methods

### 2.1. ABP design and working principles

The ABP (Implantek, Gebze, Turkey) was manufactured from a biocompatible titanium alloy (Ti6Al4V) and gathered US and EU patents (patent numbers WO2014033088A1, US9138270B2, and EP2890313B1). The ABP’s biomechanical properties were tested in vitro [15] and are given in the Table.

**Figure 1:**
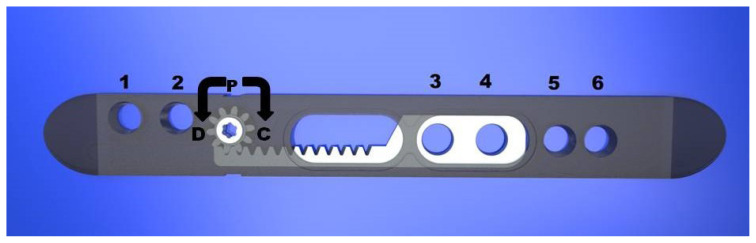
The ABP has 6 locking screw holes (numbered 1–6) and 1 pinion mechanism (P). Holes 3–4 make up the dynamic part and holes 1–2 and 5–6 constitute the static part. Rotating the pinion mechanism screw in the “D” direction results in distraction at the fracture line, while rotating in the “C” direction allows compression.

The ABP is composed of static and dynamic parts (Figure 1). The outer edge of the plate and the two sets of holes on the ends of the ABP form the static part. The dynamic part constitutes the pinion mechanism and associated screw holes. The dynamic part allows movement with the pinion mechanism and all adjustments are made using a manual screwdriver. Rotating the pinion mechanism screw 180 degrees in the “D” direction results in a 1 mm distraction at the fracture line while rotating the pinion in the “C” direction results in compression. The screw holes are similar to those of the locking screws of the LCP plate. The ABP was manufactured with 3.5 mm and 4.5 mm dimension options, and the 3.5 mm version with 6 holes was used for this study (Figure 1).

### 2.2 Segment transfer surgical technique

**Figure 2:**
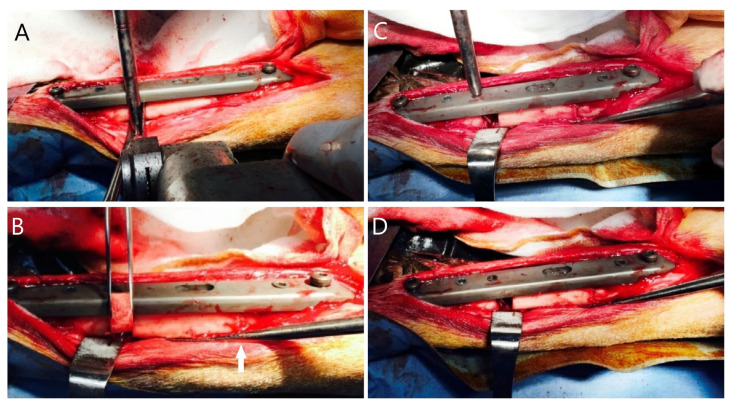
(a) The ABP was placed parallel to the long axis of the bone. The static part of the ABP was fixed to the bone through holes 1–2 and 5–6. The dynamic part of the ABP was fixed to the bone by the insertion of the special screws through holes 3 and 4. The bone defect model was created by osteotomy with a mini saw between the 2nd and 3rd holes of the ABP. (b) The white arrow indicates the osteotomy performed for the transfer of the segment between holes 4 and 5 of the ABP. (c,d) The pinion mechanism was rotated with a manual screwdriver in the “D” direction so that distraction was obtained in the osteotomy area.

**Figure 3:**
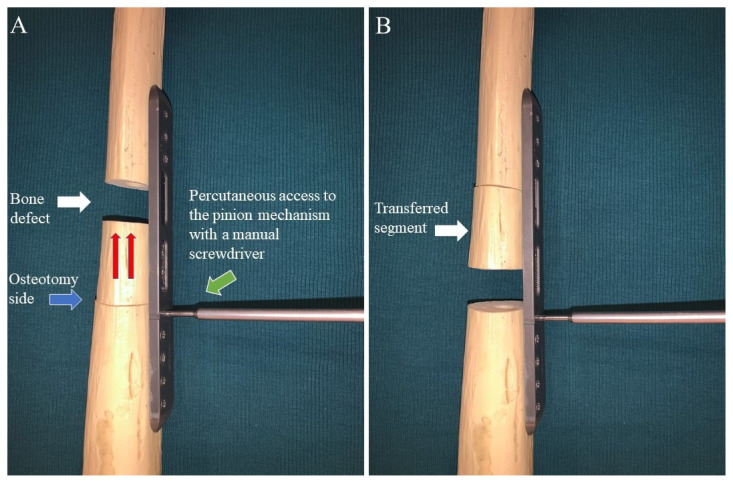
Description of ABP’s working mechanism on a saw bone model. (a) The white arrow indicates the bone defect and the blue arrow shows the osteotomy side for the segmental bone transfer. The red arrows indicate the direction of the segmental bone transfer and the green arrow indicates how to access the plate with a manual screwdriver through the percutaneous incision. (b) The successful transfer of the segmental bone (white arrow) to the bone defect.

The steps of the segment transfer surgical technique using the ABP are as follows:

1. The ABP is placed parallel to the long axis of the bone (Figure 2a).
2. The static part of the ABP is fixed to the bone at holes 1–2 and 5–6, sequentially.
3. The dynamic part of the APB is fixed to the bone with special screws through holes 3–4.
4. The bone defect is adjusted between holes 2 and 3 (Figure 2b).
5. A second osteotomy is performed to maintain the segment transfer between holes 4 and 5.
6. The pinion screw is rotated using a manual screwdriver in the “D” direction on a daily basis to maintain distraction in the osteotomy site (Figures 2c and 2d, Figures 3a and 3b).

### 2.3. Animal model

Use of the ABP was evaluated on the segmental tibia defects of sheep after getting local ethics committee approval (Approval number: 2014/7-08). The study was conducted in accordance with the Helsinki Declaration. The sheep used in the study were owned by the institution that approved the institutional review board.

At the start of each procedure, 2.5 mg/kg of Rompun and 20 mg/kg of ketamine were intramuscularly applied to the sheep for sedation and light anesthesia, and 20 mg/kg of cefazolin was used for antibiotic prophylaxis. Then, using the appropriate length of cannula, vascular access to the animal was established, and the animal was placed on the operating table. Additional drug doses required for full anesthesia were Rompun (5 mg/kg) and ketamine (50 mg/kg). The sheep was put in a prone position, the four extremities of the sheep were connected to the operating table in the appropriate surgical position, and the fur on the legs was shaved. After mechanical cleaning of the surgical site, sterility was obtained by using a povidone iodine solution. During the operation, anesthesia was maintained with additional dosing. Postoperatively, 30 mg/kg of metamizole was used for pain control. Disposable gowns, aprons, gloves, stitches, instruments, implants, and other surgical consumables were all prepared in sterile conditions.

In order to minimize the number of animals used in the study, 10 tibias of 5 sheep were operated on in the same session. The right front and left rear legs of each sheep were marked with a surgical pen before the procedure started. Following the skin incision, the subcutaneous tissues were dissected with electrocautery to control the bleeding. After the bone was exposed, the ABP was placed onto the outer surface parallel to the long axis tibia. An ABP with 6 holes was used in the present study. The static part of the ABP was fixed to the bone at holes 1–2 and 5–6. The dynamic part of the ABP was fixed to the bone by the insertion of the special screws through holes 3–4. A 15-mm-long bone defect was created with a miniature saw between holes 2 and 3 of the ABP. A second osteotomy was performed for the transfer of the segment between holes 4 and 5 of the ABP. Following the application of the ABP, the wound was closed appropriately except for the skin above the pinion mechanism, which was left open to allow percutaneous access to the plate. Starting 3 days postoperatively, daily lengthening of 1 mm was done by turning the pinion screw on the plate with a manual screwdriver through the unclosed percutaneous incision for a total of 15 days (Figures 2a–2d). Skin dressing with a povidone iodine solution was used following each lengthening process.

After 3 months of follow-up, 2.5 mg/kg of Rompun and 20 mg/kg of ketamine were intramuscularly applied to the animals for sedation and light anesthesia. After waiting for the drugs to take effect, the animals were euthanized using intracardiac high-dose sodium thiopental. Immediately after confirmation of cardiac and respiratory arrest by the veterinarian, the animals were subjected to radiological and histological examination.

### 2.4. Radiological evaluation

Radiological evaluation was performed in the 4th and 12th weeks after implantation. In order to evaluate the regeneration efficiency of the fracture site and the bone union, conventional anteroposterior and lateral graphs were performed.

### 2.5. Histological evaluation

Bone tissue samples obtained for light microscopic examination were fixed in 10% neutral formalin for a week. Then, the tissue samples were washed with running water to remove the formalin and placed in a 25% formic acid solution for 3 days. After 3 days, they were washed with running water and placed in a 0.35 M sodium sulfate solution for 3 days. At the end of this process, the tissue samples were processed using a Leica TP 1020 automatic tissue processor. The tissue pieces were embedded in paraffin after successive dehydration steps, and sections about 5 μm in thickness were obtained. Slides were dewaxed in xylene, dehydrated in graded alcohol, and stained with hematoxylin and eosin. Slide observation was performed using an Olympus BX53 light microscope.

## 3. Results

**Figure 4:**
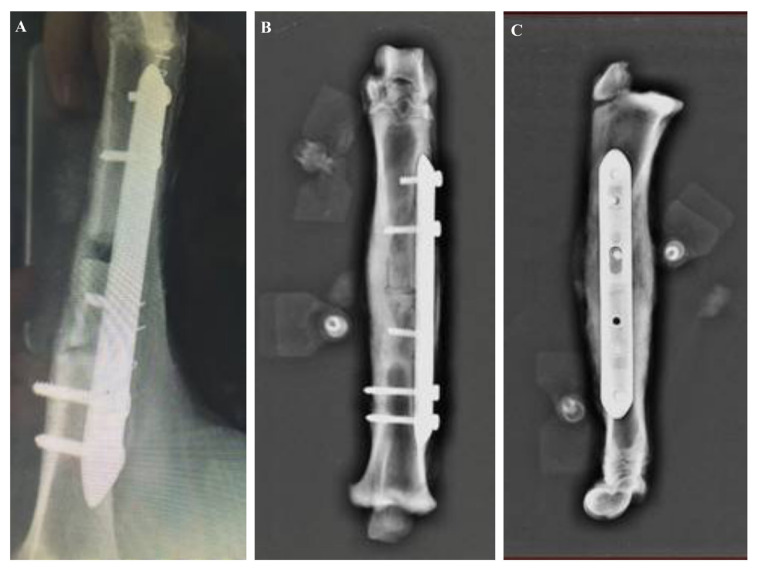
Radiographic evaluation of the surgical site at (a) 4 weeks and (b,c) 12 weeks.

Radiological evaluation of the surgical sites at 4 weeks after implantation revealed that 14.6 ± 1.2 mm of sheep tibia bone segment was completely transferred in the proximal direction throughout the defect. Complete bone healing was observed at 12 weeks along with healing of the osteotomy site (Figures 4a–4c).

**Figure 5:**
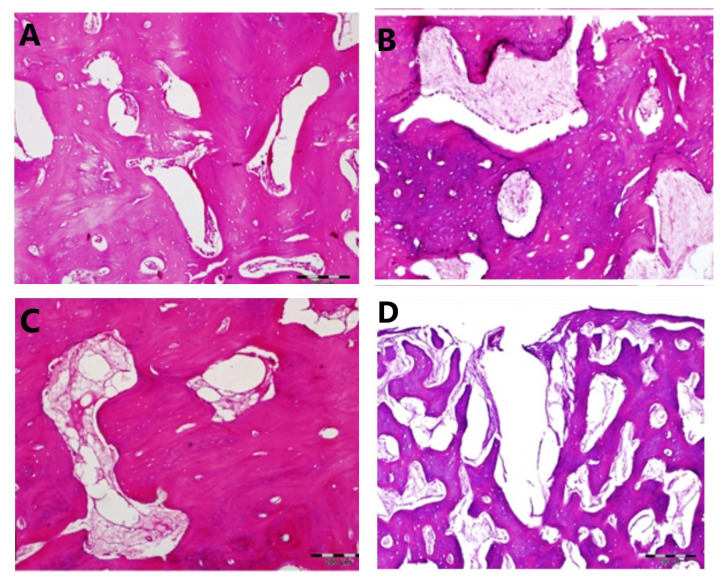
Light microscopic appearance of (a) the transferred segment, (b) the docking zone, (c) the extension zone, and (d) the screw zone. Bar = 200 μm.

In the sections obtained from the transferred segment, normal bone histology with concentric lamellae around haversian channels, interstitial lamellae between concentric lamellae, osteocytes located in the lacuna, and endosteal cells lining bone marrow spaces were clearly observed (Figure 5a). In the sections taken from where the transferred segment reached the native bone “docking zone”, fibrous tissue areas were apparent as the first stages of new bone formation and there was remarkable unorganized chondroblast hyperplasia (Figure 5b). In the sections obtained from the tissues taken from the extension region, only new bone formation was observed (Figure 5c). In the sections taken around the screw on the transferred region, new bone formation and fibrous connective tissue formation were observed. It was observed that the connection of the host bone tissue to the screw was not achieved by osseointegration but by fibrous connective tissue formation (Figure 5d).

No implant failure or failure of the lengthening mechanism was observed during the 12 weeks of follow-up. In contrast to the concerns regarding the daily opening of a wound over a plate, no surgical site infection or deep infection occurred.

## 4. Discussion

This study is the first report of using an ABP alone for the treatment of segmental bone defects in an animal model. The ABP successfully transferred 14.6 ± 1.2 mm of sheep tibial bone segment without any complications. This result validates our hypothesis that segmental bone transfer could be performed with an ABP.

The technique of distraction osteogenesis is the mainstay of limb lengthening and segmental bone transfer procedures [16]. The Ilizarov technique is one of the primary well-defined methods of distraction osteogenesis. In this technique, fixation is performed using an inserted frame with external wires and pins before the metaphyseal osteotomy. After the latency period, the bone segment is generally shifted by 1 mm daily. When the shifted segment reaches the docking site, it is compressed and held in position until union is achieved. This consolidation phase may require a prolonged period, so pin tract infection is the main complication of this technique and its risk is increased with the duration of external fixator [17]. Additionally, in some instances, a prophylactic fixation with a plate and screw is required to protect the consolidation callus after removal of the external fixator [18]. Loss of axial alignment during the distraction period is not an uncommon situation when using the external fixator alone. For this reason, intramedullary nails are used alone or in combination with external fixators. Intramedullary nails are reported to decrease the length of time an external fixator is needed, and they also assist in maintaining alignment [19]. Intramedullary nailing with an external fixator is technically demanding because the intramedullary nail reduces the space available for optimal fixation of pins and wires. While their combination decreases the time that external fixation is needed, which can decrease the risk of pin tract infection, it may cause more serious intramedullary infection due to the proximity of the pins and nail [20]. In the pediatric population, application of intramedullary nails is limited because of open growth plates [21]. This is an important limitation because treatment of LLD is recommended to be done in early childhood [22]. Intramedullary nails cannot be used if the bone does not have adequate intramedullary space for the nail, so the use of intramedullary nails and external fixators is not suitable for distraction osteogenesis, especially in the upper extremities [23]. The new design of a motorized intramedullary nail potentially resolves the simultaneous need of an external fixator; however, this approach has its own problems, like failure in the lengthening mechanism, migration of the locking screws, and intramedullary infections [12].

The ABP may solve the Ilizarov technique’s key problem of pin tract infection, which was reported to occur in 10%–20% of cases [11]. The ABP is a self-internal splint which does not require a secondary implant or procedure until consolidation is achieved. The ABP is a user-friendly device that protects the axial alignment of the bone during distraction. We believe that there is a gap in the literature regarding the optimal implant for upper extremity distraction osteogenesis [23] and pediatric bone lengthening [24]. The ABP can fill this gap in the upper extremity with its extramedullary design that eliminates the neurovascular complications of external fixators. In the pediatric population, the ABP can be used safely without interfering with the growth plates. On the other hand, the ABP has a few limitations. The ABP achieves distraction osteogenesis through segmental bone transfer, whereas both the expandable nail and external fixator achieve this by distraction of the main bone fragments without reliance on the availability of a bone segment for transfer. Furthermore, due to the fixed length of the ABP’s static part, the desired distraction distance is set at the time of surgery and cannot be changed during treatment.

The ABP requires daily screwdriver access to the pinion mechanism screw hole through an unclosed skin incision, which carries the potential for infection. However, we did not observe any wound infection or deep infection during follow-up observations. This may be due to the subcutaneous nature of the tibia of the sheep allowing easy access to the ABP. We acknowledge that the current format of the ABP requiring percutaneous access is prone to postoperative infections and poses a big disadvantage for human use. Nonetheless, our primary aim was to demonstrate a novel method of segmental bone transfer over a plate in vivo. We believe that the successful application of the ABP for the segmental bone transfer provides evidence for the usefulness of more advanced plate designs, such as a remote-controlled ABP. We also think that these advanced plate designs would be more beneficial for human use.

Our study had some limitations. Since the study was performed on sheep tibia, the application of this plate in clinical practice requires meticulous consideration. Furthermore, there was no control group with a different implant, so we can only compare our results with earlier research. No biomechanical tests of the explanted tibia were performed.

## 5. Conclusion

In conclusion, the ABP can be an option for segmental bone transfer. It may bring the advantages of intramedullary nails and external fixators while reducing their potential complications. Its unique design not only allows distraction at the osteotomy site but also has the ability of compression. It is thought to be useful in fracture treatment by using its adjustable compression utility, but new studies are needed to show its efficacy in a fracture model.

## Figures and Tables

**Table:** Biomechanical properties of the ABP [15].

Density: 4429 kg/m^3^
Young’s modulus: 113.8 GPa
Poisson’s ratio: 0.342
Yield strength: 790 MPa
Ultimate tensile strength: 860 MPa
Bending stiffness: 1775 N/mm
Fatigue life at alternating 2300 N: 10^6^<
Fatigue life at alternating 3500 N: 48.7
